# Supra-Descemet’s Fluid Drainage with Simultaneous Air Injection: An Alternative Treatment for Descemet’s Membrane Detachment

**DOI:** 10.4103/0974-9233.80712

**Published:** 2011

**Authors:** Alireza Ghaffariyeh, Nazafarin Honarpisheh, Tooraj Chamacham

**Affiliations:** Department of Ophthalmology, Dr. Khodadoust Eye Hospital, Shiraz, Iran

**Keywords:** Air Injection, Descemet’s Membrane Detachment, Supra-Descemet’s Fluid Drainage

## Abstract

In this report, we present an alternative technique to manage Descemet’s membrane detachment (DMD). We call the technique supra-Descemet’s fluid drainage with intracameral air injection. Under topical anesthesia, we injected air through the stab incision to fill 2/3 of the anterior chamber. Then we inserted the tip of a curved 10/0 needle through the corneal surface (entry angle at 45 degrees) into the supra-Descemet’s area 3 times to drain this fluid. In our method, we neither injected expanding gas or viscoelastic nor used a suture. Consequently, there was little chance for suture-induced astigmatism or increased intraocular pressure. This technique may be considered a relatively safe and simple surgical method for the management of postoperative DMD.

## INTRODUCTION

Descemet’s membrane detachment (DMD) can be triggered by any factor causing the separation of Descemet’s membrane (DM) from the overlying stroma.

Various factors can contribute to the formation of DMD, such as congenital factors (ie, keratoconus, keratoglobus, and congenital glaucoma), surgical factors (ie, penetrating keratoplasty, full-thickness lamellar keratoplasty, trabeculectomy, iridectomy, cyclodialysis, viscocanalostomy, holmium laser sclerostomy, and especially cataract surgery, anatomical factors, eye trauma, and chemical burns.^1^–^4^

DMD has been divided into 2 groups:

Planar DMD where DM separation from its overlying corneal stroma is less than 1 mm^1^ ; and nonplanar DMD where DM separation from its overlying corneal stroma is greater than 1 mm.^1^

Each of these groups has been divided into 2 subgroups (peripheral and combined DMD). In peripheral DMD, the detachment is merely confined to the peripheral cornea, but in combined DMD the detachment involves both peripheral and central cornea.^1^ Cataract surgery has been reported to be the most common cause of DMD, most probably due to the recent significant rise in the number of clear-corneal cataract operations, blunt instruments, and poor technique.^2^

In this article, we report a case that was referred to our center with DMD 1 week after undergoing phacoemulsification cataract surgery. We treated the DMD by performing our suggested surgical method.

### Technique

A 75-year-old female patient was referred to our center for DMD in her right eye. She had undergone phacoemulsification cataract surgery with clear-corneal temporal incision 1 week before referral. She had neither a history of eye trauma nor ocular or medical disorders.

She had undergone an air injection for her DMD 1 day after the first operation, which failed.

On examination, her uncorrected visual acuity was 20/100. Slit-lamp examination of the right eye revealed a combined nonplanar DMD in temporal incision, involving about one-third of the corneal surface with central extension, as well as mild corneal edema. Intraocular pressure (IOP) in her right eye was 17 mmHg. Initially, medical therapy was instituted consisting of chloramphenicol 0.5% every 4 h and prednisolone acetate 1% every 2 h.

**Figure 1:**
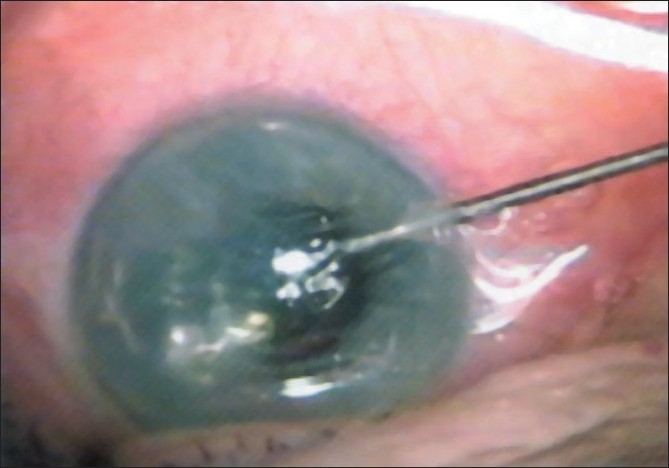
Injecting air through the previous stab incision to fill 2/3 of the anterior chamber

**Figure 2:**
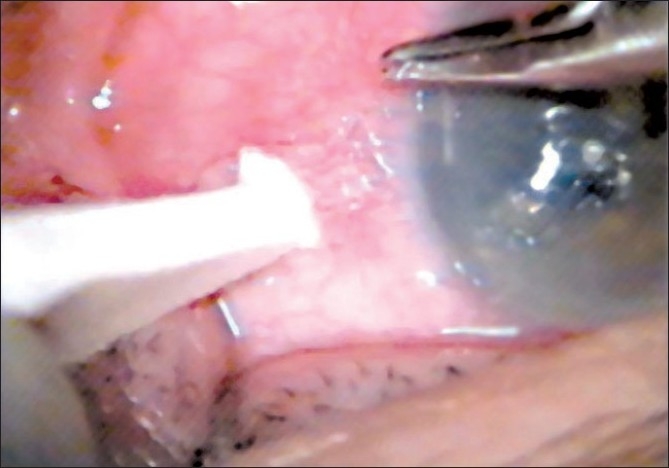
Inserting a needle of 10/0 suture with an angle of 45 degrees to supra-Descemet’s area

**Figure 3:**
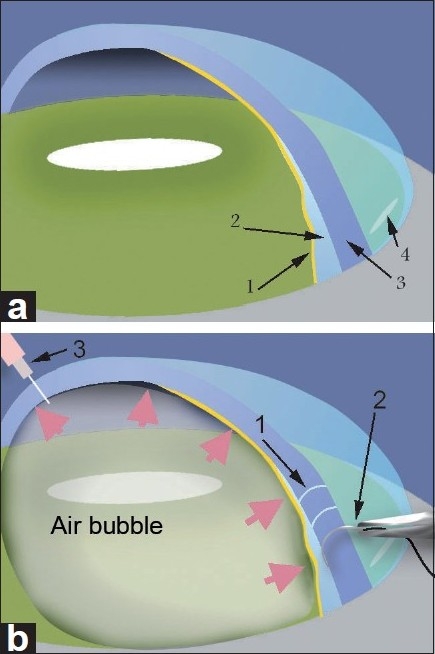
Schematic illustration of the technique. (a) The presence of Descemet’s membrane (DM) detachment and fluid between the DM and posterior stroma. 1: DM; 2: supra-Descemet’s fluid; 3: corneal stroma; 4: clear-corneal temporal incision. (b) 1: Needle tracts in the corneal helps in the drainage of the fluid to the corneal surface; 2: the tip of a curved 10/0 needle was inserted through the corneal surface at a 45 degree angle into the supra-Descemet’s area in the detached area; 3: air bubble being injected into the anterior chamber through stab incision.

On the day of admission, supra-Descemet’s fluid drainage with intracameral air injection was performed. Under topical anesthesia, air was injected through the previous 3 o’clock stab incision to fill 2/3 of the anterior chamber to limit the detached area [Figure 1]. The tip of a curved 10/0 needle was then inserted through the corneal surface at a 45 degree angle into the supra-Descemet’s area in the detached zone from center to periphery 3 times until the fluid appeared in the corneal surface [Figure 2]. When the corneal stromal resistance finished, we stopped needle tip insertion. A clear fluid appeared on the corneal surface, while entering the supra-Descemet’s area showing successful drainage of the fluid. If no fluid appeared on the corneal surface, the procedure can be repeated several times. The needle was moved side to side to enlarge the needle tract for future drainage [Figure 3].

**Figure 4:**
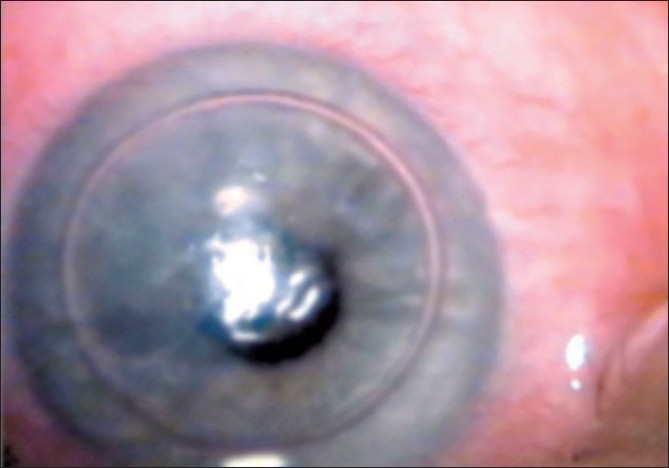
Following fluid drainage, the patient placed in a reclining position for 1 hour

After performing the procedure, drops of the drained fluid appeared on the corneal surface similar in appearance to dew on a leaf, indicating the successful supra-Descemet’s fluid drainage. Following fluid drainage, the patient was placed in a reclining position for 1 h Figure 4

Postoperative slit-lamp examination revealed that the DM was successfully reattached. One day later her uncorrected visual acuity was 20/40, IOP was18 mmHg and the corneal edema had resolved.

One week later her uncorrected visual acuity had improved to 20/30. By 2 months postoperatively, her cornea was completely clear and her uncorrected visual acuity had further increased to 20/25.

## DISCUSSION

As the natural history of DMD has not been completely elucidated, the suitable timing for intervention remains unknown. Planar detachments are more likely to resolve spontaneously.^5^ Since persistent DMD can cause corneal edema and visual loss, early interventions for nonplanar DMD has been generally recommended.^1^,^5^–^7^

A few studies have reported spontaneous resolution of large persistent nonplanar DMDs. One such example was reported by Watson *et al*.^5^ For managing an intraoperative large DMD, Walland **et al**.^6^ have suggested the use of an expanding gas. The intracameral injection of an expanding gas (eg, sulfur hexafluoride) has shown to be an effective treatment, but not in all cases of DMD.^2^

Menezo *et al*.^7^ have previously suggested a treatment method for DMD by filling the anterior chamber with air after a 3 port paracentesis aimed at draining the sub-DM fluid. Menezo *et al*. reported a successfully reattached DM and good visual outcome.^7^

Fluid drainage between the DM and the corneal stroma may have been critical to the success of the reattachment. The first attempt to reposition the DM in this case with air tamponade failed, because the entrapped fluid between the DM and the corneal stroma was not successfully drained. In these cases, the air bubble must be large enough to push DM up against the stroma, and allow the endothelium to pump it into place for proper adherence.

A detached DM that is scrolled, shredded, or severely damaged is unlikely to be repaired using this technique and may require more delicate surgical manipulation.

In our case, the DM wrinkles, haze, or opacity, which sometimes occurs after a successful reattachment, was not seen. The formation of Descemet’s wrinkles can result in irregular astigmatism.^1^,^5^

In our suggested technique for the treatment of DMD, minimal instrumentation is required. Additionally, we neither injected expanding gas or viscoelastic nor required a suture. Consequently, there was little chance for suture-induced astigmatism or increased intraocular pressure.^1^

The presented case responded to this intervention very well and the corneal edema fully resolved. We believe that this is a relatively safe and simple method for the surgical management of DMD and can be considered as an effective alternative technique.
